# Clinical Lecture*Delivered at the Medico-Chirurgical Hospital of Philadelphia, Pa.

**Published:** 1897-02

**Authors:** John V. Shoemaker

**Affiliations:** Professor of Skin and Venereal Diseases in the Medico-Chirurgical College and Hospital of Philadelphia


For the Texas Medical Journal.

### CLINICAL LECTURE.*

### Secondary Syphilis—Varicose Veins and Eczema—Chancroids—Dermatitis Venenata.

BY JOHN V. SHOEMAKER, M. D., LL. D.,
Professor of Skin and Venereal Diseases in the Medico-Chirurgical College and Hospital of Philadelphia.

### SECONDARY SYPHILIS.

THE first patient whom I bring before you this morning is a young woman, 21 years of age, who gives the following history: Three weeks ago she was delivered of an apparently healthy child, whose body was free from blemish. On the day succeeding her confinement, a rash appeared upon her left forearm. The eruption consisted of small papules, which itched and smarted, especially when brought in contact with water. At nearly the same time lesions of the same character developed upon the right forearm. Two or three days later papules came out upon the face, first upon the forehead, and then upon the nose, cheeks and chin.

The papules are confluent, of a dull, red color, and many, particularly upon the face, are covered with scales, or thin crusts. In the early evolution of the rash the eyelids were swollen. A few papules occurred along the margin of the lids and behind the ears. There have been none upon other parts than those mentioned.

Three months ago the patient had a single sore upon the vulva, deeply ulcerated, about the size of a five-cent piece in diameter. It furnished a moderately free discharge, and was quite painful. The patient is not aware that there were any glanular enlargements in either groin.

For about a week before the birth of her child she suffered from a more or less continuous headache, had fever, sore throat, and the cervical glands were enlarged. There has been some falling of the hair.

The foregoing history, though not as exact in all points as we might wish, is a fair sample of what we generally obtain from this class of patients. The symptoms preceding the outbreak

---

*Delivered at the Medico-Chirurgical Hospital of Philadelphia, Pa.

of the rash are very suggestive. The fever, she states, was moderate, and unaccompanied by backache. She did not suffer from bone-pains in the arms or legs. The sore throat lasted about two days. The continuance of the constitutional symptoms was about a week prior to the evolution of the rash. The itching which attended the first manifestation of the eruption speedily disappeared. The lesions were at first distinctly papular before any scaling took place. The rash is, beyond question, the small papular syphiloderm. I had occasion not long ago to speak of the significance of frontal headache, occurring as a prominent symptom ushering in the secondary stage of syphilis. In many such cases it is difficult or impossible to discover the initial lesion unless the patient is seen early and most carefully examined. The primary sore may escape observation. Nevertheless, with such a history in a young woman,—a persistent frontal headache, a febrile condition, a sore throat and thinning of the hair,—we have strong reasons to suspect the presence of syphilis before the advent of the rash. Again, as regards the lesions themselves in this case, they consist of small papules, congregated in groups, of a peculiar color, and, as they are in a state of retrocession, they have become covered with scales.

This form of syphiloderm bears a certain degree of resemblance to papular eczema, but the points of distinction between the two eruptions are sufficiently distinct. As a rule, the rash of eczema seeks the flexor surfaces, occurs in the flexures of the elbows and knees, or upon the trunk. The papules of eczema are smaller than those of the case before us, they are more closely approximated to each other, and they are accompanied by more irritation of the surface. This, like all the other forms of eczema, with the exception of the erythematous, is accompanied by more or less moisture. Itching is constant, and causes great distress. The patient can scarcely abstain from scratching, and the affected surface is often torn by the nails. This variety rarely attacks the face. When it occurs upon the face, eczema is generally either erythematous or vesicular.

The small or miliary papular syphilide is one of the earlier manifestations of the secondary stage. Itching is an unusual symptom in syphilitic eruptions of any kind, although it may occur in exceptional cases, and at an early period of the evolution of the exanthem. It is a never prominent feature of the case, and soon disappears.

As regards treatment, I shall direct this patient to take three grains of mercury with chalk four times a day. Her mucous membranes are in a deplorable condition,—the tongue large, flabby, and covered by considerable exudation. There is inactivity along the entire *primæ viæ*. In such a case the iodide of mercury or potassium may not be absorbed. The remedy capable of acting upon the glanular system in general, and the liver in particular, is the combination of calcium and mercury which I have named. This is a very valuable preparation under these circumstances, and especially in cases where we cannot make use of the hypodermic method. No local treatment is needed.

### VARICOSE VEINS AND ECZEMA.

II. Our next patient is a woman, 55 years of age, who suffers from engorged, varicose veins of the legs. This is quite a common affection in elderly persons, and is especially frequent in women. It very often dates from the later months of gestation. The enlarged womb, pressing upon the iliac veins, obstructs the return of blood from the extremities. As a consequence, the veins passively dilate, their lining membrane is altered, the valves become insufficient, shorten or break down, and are unable to support the column of blood. Once inaugurated, the process tends to go on from bad to worse. In multiparæ, it is not infrequent to see a varicose condition of the veins of the leg, thigh and vulva. Any condition which produces difficulty in the venous circulation may be a cause of varicose veins. Thus, the use of tight garters, the presence of pelvic or abdominal tumors, certain diseases of the heart or lungs, which retard the circulation, may occasion dilation and varicosity of the veins. Occupations which necessitate being constantly on the feet tend to produce this disease of the veins. It sometimes occurs as a sequela of typhoid fever, on account of debility of the nervo-muscular system, and of the heart. Varicose veins may, of course, occur in other situations than upon the lower limbs. These are, however, the parts usually involved, since they are most frequently exposed to the operation of causes which embarrass the circulation, and since in them the blood must be propelled in opposition to the force of gravity.

Dilated, engorged and tortuous veins entail further consequences. The outer coat of the vessels is thickened, or, in some instances, thinned. Interference in the circulation diminishes local nutrition, and, accordingly, the occurrence of eczema or ulcer of the leg is no uncommon complication.

In the woman before us, we find an eczema as a result of the condition of the veins. Varicose ulcers, as they are termed, are quite frequently met with among dispensary patients.

Another consequence of varicose veins, in old cases, forms an emergency case which any one of you is liable to meet with in his first day of practice. I refer to hemorrhage. An attenuated vessel may burst, and a large quantity of blood may be rapidly lost. I have seen patients rapidly exsanguined by this accident, and death has sometimes occurred before efficient help could be rendered.

Our present patient seems to enjoy good general health. Her tongue is clean and bowels regular. She complains that she has some rheumatic symptoms from time to time.

This patient shall be given a remedy which, by virtue of its beneficial effect upon the muscular coat of the veins, is peculiarly adapted to be used when these vessels are enfeebled and varicose. Clinical experience has shown that hamamelis possesses marked efficacy in this class of cases. The fluid extract is probably the best preparation, and in this case we will order:

℞ Extract hamamelis, fl.
Glycerine............................aa f℥j

M. Sig: One or two teaspoonfuls three times a day.

Externally, on account of the eczematous condition, the following ointment will be applied:

℞ Pulv. camphor.......................gr. x
Acid salicylic........................gr. xx
Acid carbolici.............................M x
Sulphur sublimat...................℥ss
Ung. zinci oxide.....................
Ung. aquæ rosæ.................. ...aa ℥ss

M. Ft. ungt.

In addition to these measures, the patient shall be directed to keep the limb bandaged. The ordinary muslin roller-bandage is used among poor patients. The Martin rubber bandage, or a well made elastic stocking, answers the purpose more effectually by affording a continuous gentle support and promoting circulation through the healthy deeper veins instead of the weakened superficial vessels. Various surgical procedures are also sometimes practiced in order to secure obliteration of the diseased veins.

CHANCROIDS.

III. A lusty young colored man, 23 years of age and a gardener by occupation, came to the Clinic a few days ago on account of the sore and swollen condition of the penis. In his history he stated that he had first noticed sores upon his virile organ about ten days ago. He had not indulged in sexual connection for a month. The sore was situated upon the inner surface of the prepuce, and toward the left side. It was small and ulcerated, but was unaccompanied by much secretion. It increased in size, and in the course of the next three days the sore began to furnish a discharge and the prepuce to swell. There has been no discharge from the urethra or pain on passing urine. At the time of his first visit there was considerable tumefaction, and the foreskin could be retracted only for a very short distance. Although but a small portion of the glans in the vicinity of the meatus could be exposed, several small ulcers could be discerned within that limited space, some being situated upon the margin of the prepuce and others upon the glans.

The glands in each groin are somewhat enlarged, hard and slightly tender upon pressure, especially upon the left side. There have been no constitutional symptoms in the case. The prepuce has been slit up, exposing to view the entire glans penis. A number of ulcerated lesions were found upon the foreskin, glans and frenum.

With the foregoing history in mind and the patient before us, let us now inquire as to the nature of the sores with which he is afflicted. No other origin than venery can be assigned. From the number of the lesions, the pain to which they gave rise and the absence of induration at their bases, the presumption here is that we have to deal with chancroids. On the other hand, if we may accept the patient's statement implicitly the length of the incubation period and the enlargement of the inguinal glands point rather to chancre than chancroids. It is customary to say that the chancroid has no period of incubation, i. e., the lesion appears very rapidly after the infecting intercourse, showing that the action of the virus upon the tissues is immediate. Under favorable circumstances, where there has been an abrasion, absorption takes place at once, and a chancroid may develop within twenty-four hours. When the epithelium is intact a longer period is required, but in the vast majority of cases the sore appears within a week after exposure. In a few instances, according to the statistics of distinguished authorities, a period of two weeks, three weeks, or even longer, may elapse before a sore occurs. This may be one of those exceptional cases. Again, when an illiterate man or woman asserts that a particular event occurred "about a month ago," a wide margin is left for doubt as regards the exact date. Sores, also, may have existed for several days before they were observed by the patient. For these reasons, it is possible that, although the stage of incubation has been unusually long, we are not obliged, on that account, to withdraw the diagnosis of chancroid.

The ganglionic enlargements of the groins bear more resemblance to those attendant upon syphilis than to the buboes of chancroid. The latter usually affords an excellent illustration of acute inflammation of a high degree of intensity. There is much swelling, decided heat and pain, notable interference with locomotion, and the process terminates in an abundant production of pus. These are, indeed, the characteristic signs of typical chancroid bubo, and when present can be overlooked by neither patient nor physician. There are, however, exceptions to every rule. The occurrence of an inflammatory, supurating bubo is by no means an invariable complication or sequence of chancroid. It seems probable that in this case the virus has excited but a moderate degree of adenitis. For the gross appearance of the sores is not denotive of chancre. The initial lesion of syphilis is almost always solitary. Multiple chancres are sometimes, but rarely, observed. Whereas, chancroids are, as a rule, numerous. The pus furnished by one sore inoculates a surrounding spot and produces a second lesion of the same kind, which in its turn, if left to itself, forms the starting point of a third chancroid, and thus the process continues. This feature of auto-inoculability, as it is termed, is distinctive of chancroid as opposed to chancre. We, therefore, look upon his case as one of chancroids. Scrupulous cleanliness is a prime essential in the treatment of these cases. The affected surfaces must be frequently cleansed with warm water, which may be serviceably medicated with carbolic acid, Labaraque's solution, etc., and the sores, in the interval, kept separated in such a manner that they may not contaminate the adjacent parts. Cauterization is generally effectual in checking the progress of the disease. Dry antiseptic dressings are likewise beneficial in most cases. In the patient before us we shall make use of a dusting powder which has been found very useful in our service, and which consists of one part of calomel to three parts of acetanilid.

DERMATITIS VENENATA.

IV. This young man, 22 years of age, came to the Clinic on account of an eruption upon the hands and foreams. He is an electro-plater by occupation, and in the seven years during which he has been engaged in that work, has suffered from four or five attacks similar to the present. A part of his business consists in making the cyanide solution employed in electroplating. Between three and four weeks ago, when preparing a fresh solution, some of the liquid splashed upon the fingers of the right hand. On the following day the fingers were rough and itched. Minute red papule's, about the size of a pin's head, next formed upon the affected surfaces. At the end of a week the papules were converted into vesicles and, as these ruptured, considerable "weeping" occurred. From the fingers the disease spread to the hand and forearm. The affection was transferred in the same order to the left hand and forearm. The last-named regions, he believes, were involved through contact with the serous discharge, as he does not think that they were touched by the solution which originated the trouble. In the same manner the disease has been transferred to the forehead and the back of the ears. He states that the lips have also been affected. Upon examining the parts I find papules and vesicles seated upon a red, inflamed, thickened surface, and involving the various regions which have been mentioned. The backs of the hands and fingers are almost completely covered. In the upper part of the posterior surface of the right forearm there is some scaling. On the anterior surface the inflammation is, for the most part, dry, but at the middle there is some vesiculation with escape of fluid. Upon the left hand and forearm the aspect is similar and exhibits erythema, papules and vesicles. All of the vesicles are very small, some are intact, while others have ruptured.

The eruption has caused much smarting and itching. During the day he endeavors, with some success, to control his impulse to scratch, but at night he wakes from his sleep to find himself tearing at the diseased parts. By this means the vesicles are broken and the inflammation maintained. The patient says he has noticed that if his general health is depressed, or if he is constipated, he is particularly liable to acquire the disease. This is also the observation of all of his fellow workmen. He complains also of pain and a sense of weight in the left forearm.

This is a case of dermatitis produced by contact with an irritant chemical solution. The effect is due to potassium cyanide alone, as the fluid contained no silver. The inflammation involves the different layers of the integument, and manifests itself in the various lesions perceived in this patient. This man must discontinue his work. He shall be given internally:

℞ Sp. ammon aromat................ m cc
Aloin................................. gr. iii
Tr. cardamom co.................. f ʒi
Aq. cinnamon q. s. ad............ f ʒv

M. Sig.: Two teaspoonfuls three times a day.

Externally:—

℞ Hydrarg. ammoniat............... gr. x
Acid, carbolici..................... mx
Pulv. camphor...................... gr. v
Urg. zinci oxid benzoat......... ʒi

M. ft. ungt.

When this ointment causes irritation sulphur may be substituted for the ammoniated mercury.

